# Diaqua­bis­{4-[(pyridin-2-yl)methyl­idene­amino]­benzene­sulfonato-κ^2^ *N*,*N*′}nickel(II) tetra­hydrate

**DOI:** 10.1107/S1600536812022179

**Published:** 2012-05-26

**Authors:** Chao-Zhu Li, Xue-Ren Huang

**Affiliations:** a College of Chemistry and Chemical Engineering, Qinzhou University, Qinzhou, Guangxi 535000, People’s Republic of China

## Abstract

In the title complex, [Ni(C_12_H_9_N_2_O_3_S)_2_(H_2_O)_2_]·4H_2_O, the Ni^II^ ion is coordinated by four N atoms from two bidentate chelating 4-[(pyridin-2-yl)methyl­idene­amino]­benzene­sulfonate ligands and two O atoms from *cis*-related water mol­ecules in a slightly distorted octa­hedral environment [Ni—N = 2.071 (3)–2.121 (3) Å and Ni—O = 2.071 (2) and 2.073 (3) Å]. In the crystal, the coordinated water mol­ecules and the four water mol­ecules of solvation are involved in inter­molecular O—H⋯O hydrogen-bonding inter­actions with water and sulfon­ate O-atom acceptors, giving a three-dimensional framework structure.

## Related literature

For the synthesis of the ligand, see: Casella & Gullotti (1981 ▶). For the synthesis, structures and applications of similar complexes, see: Zhang *et al.* (2007 ▶, 2008 ▶). For the structures of the mainly tridentate complexes with the title ligand and similar ligands, see: Correia *et al.* (2003 ▶); Jiang *et al.* (2006 ▶); Ou-Yang *et al.* (2008 ▶); Li *et al.* (2006 ▶); Huang *et al.* (2009 ▶).
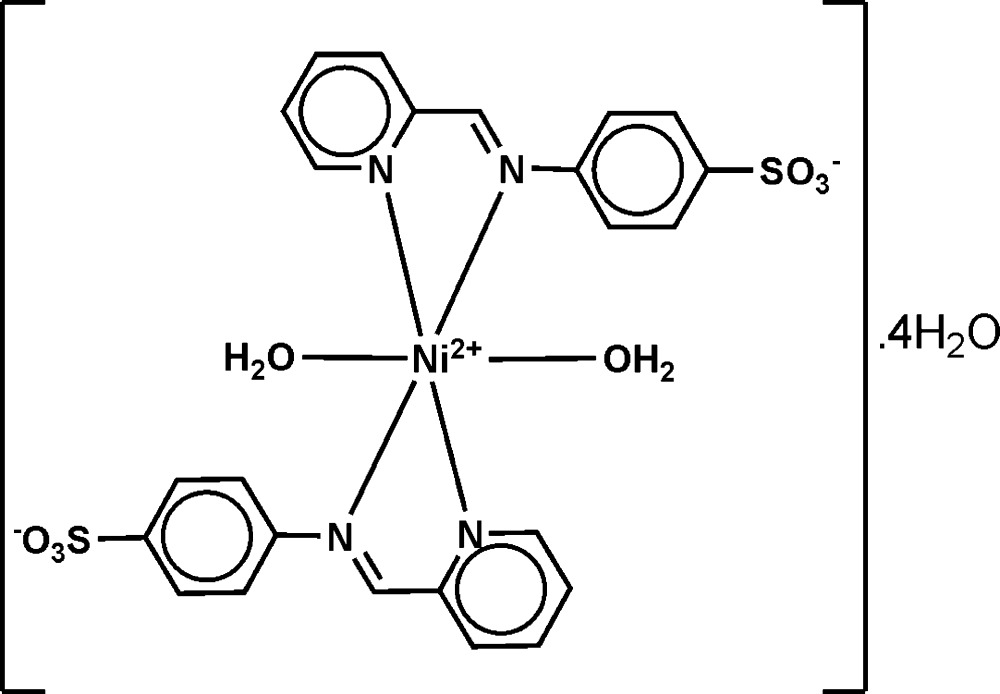


## Experimental

### 

#### Crystal data

[Ni(C_12_H_9_N_2_O_3_S)_2_(H_2_O)_2_]·4H_2_O
*M* *_r_* = 689.35
Orthorhombic, 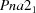

*a* = 13.865 (2) Å
*b* = 11.5310 (18) Å
*c* = 18.860 (3) Å
*V* = 3015.3 (8) Å^3^
*Z* = 4
Mo *K*α radiation
μ = 0.85 mm^−1^
*T* = 296 K
0.36 × 0.19 × 0.14 mm

#### Data collection

Bruker SMART CCD area-detector diffractometer
Absorption correction: multi-scan (*SADABS*; Bruker, 2000 ▶) *T*_min_ = 0.821, *T*_max_ = 0.889
16541 measured reflections
5315 independent reflections
4983 reflections with *I* > 2σ(*I*)
*R*_int_ = 0.028

#### Refinement

*R*[*F* ^2^ > 2σ(*F* ^2^)] = 0.034
*wR*(*F* ^2^) = 0.100
*S* = 0.96
5315 reflections
388 parameters
1 restraint
H-atom parameters constrained
Δρ_max_ = 0.44 e Å^−3^
Δρ_min_ = −0.20 e Å^−3^
Absolute structure: Flack (1983 ▶), 2540 Friedel pairs
Flack parameter: 0.00 (1)

### 

Data collection: *SMART* (Bruker, 2000 ▶); cell refinement: *SAINT* (Bruker, 2000 ▶); data reduction: *SAINT*; program(s) used to solve structure: *SHELXS97* (Sheldrick, 2008 ▶); program(s) used to refine structure: *SHELXL97* (Sheldrick, 2008 ▶); molecular graphics: *SHELXTL* (Sheldrick, 2008 ▶); software used to prepare material for publication: *SHELXTL*.

## Supplementary Material

Crystal structure: contains datablock(s) I, global. DOI: 10.1107/S1600536812022179/zs2208sup1.cif

Structure factors: contains datablock(s) I. DOI: 10.1107/S1600536812022179/zs2208Isup2.hkl

Additional supplementary materials:  crystallographic information; 3D view; checkCIF report

## Figures and Tables

**Table 1:** Hydrogen-bond geometry (Å, °)

*D*—H⋯*A*	*D*—H	H⋯*A*	*D*⋯*A*	*D*—H⋯*A*
O1—H1⋯O4^i^	0.85	1.92	2.748 (4)	166
O1—H1*A*⋯O7^ii^	0.85	2.08	2.876 (4)	156
O2—H2*A*⋯O1*W*	0.85	1.94	2.752 (4)	159
O2—H2*B*⋯O3*W*^iii^	0.85	1.82	2.659 (4)	171
O1*W*—H1*WA*⋯O4*W*	0.85	2.00	2.804 (5)	156
O1*W*—H1*WB*⋯O2*W*^iv^	0.85	1.90	2.733 (5)	167
O2*W*—H2*WA*⋯O3^v^	0.87	2.13	2.833 (5)	137
O2*W*—H2*WB*⋯O7^vi^	0.86	2.29	2.874 (5)	125
O3*W*—H3*WB*⋯O6^v^	0.85	2.00	2.813 (6)	160
O3*W*—H3*WA*⋯O5^vii^	0.85	2.15	2.930 (5)	152
O4*W*—H4*WB*⋯O8^vi^	0.85	2.17	2.846 (5)	136
O4*W*—H4*WA*⋯O5^viii^	0.85	2.06	2.903 (5)	169

Symmetry codes: (i) 
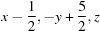
; (ii) 
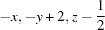
; (iii) 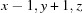; (iv) 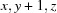; (v) 
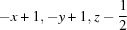
; (vi) 
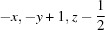
; (vii) 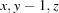; (viii) 
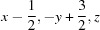
.

**Fig. 1.**
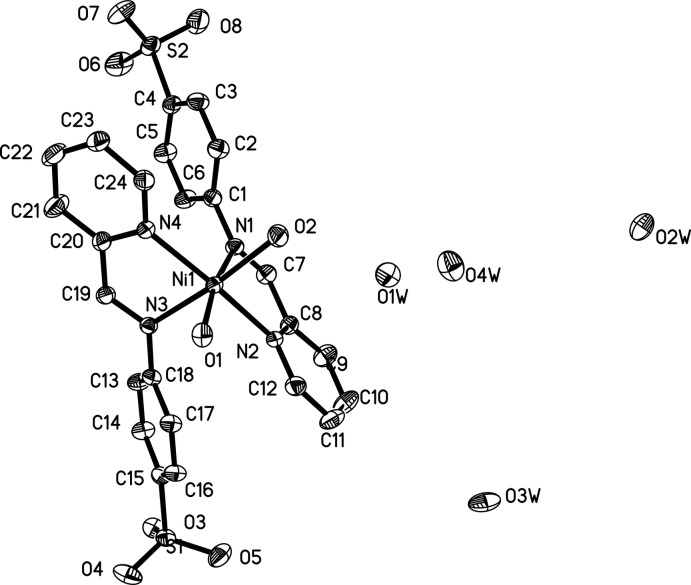
The molecular structure of the title complex, showing the atom-numbering scheme. All H atoms have been omitted for clarity.

**Fig. 2.**
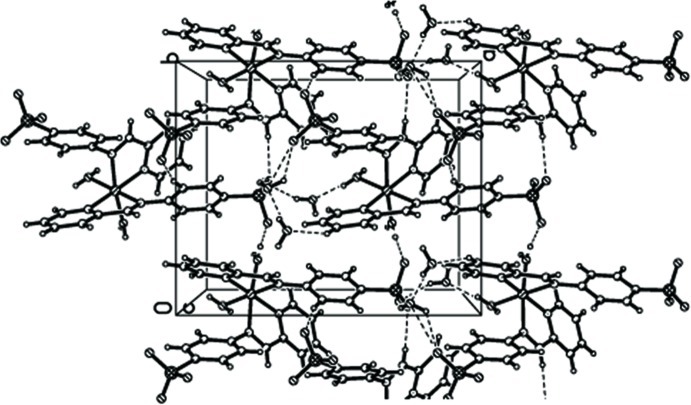
The packing of the title complex, showing the two-dimensional sheet structure in the ac plane.

## supplementary crystallographic information

### Comment

The design and control of supramolecular coordination complex networks in which both coordinate bonds and hydrogen bonds take part in the molecular self-assenbly, have recently attracted increasing interest. Schiff base complexes that contain both sulfur and amino acid functionalities have also received much attention due to their potential application in pharmacy (Jiang *et al.*, 2006; Zhang *et al.*, 2007, 2008). We report here the synthesis and the stucture of the mononuclear Ni^II^ complex with the potentially tridentate monoanionic ligand from the acid (4-pyridylmethylimine)benzenesulfonic acid (BfbaH), the title complex [Ni(C_12_H_9_N_2_O_3_S)_2_(H_2_O)_2_] . 4H_2_O. Other complexes with this ligand or similar ligands in the *N*,*N*',*O*-tridentate mode are known (Correia *et al.*, 2003; Li *et al.*, 2006; Huang *et al.*, 2009; Ou-Yang *et al.*, 2008).

The asymmetric unit of the title complex (Fig. 1) comprises a Ni^2+^ cation, coordinated by four N atoms from two bidentate Bfba ligands and two O atoms from *cis*-related water molecules in a slightly distorted octahedral environment [Ni—N, 2.071 (3)–2.121 (3) Å; Ni—O, 2.071 (3), 2.079 (3) Å], together with four water molecules of solvation. The two ligands are conformationally different [dihedral angles between the pyridine and benzene rings in each: 55.85 (18) and 43.2 (2)°]. In the crystal structure, the coordinated water molecules and the water molecules of solvation are involved in intermolecular O—H···O hydrogen-bonding interactions with water and sulfonate O-atom acceptors (Table 1) giving a three-dimensional framework structure (Fig. 2).

### Experimental

The potassium salt of (4-pyridylmethylimine)benzenesulfonic acid (BfbaK) was synthesized according to the literature method (Casella & Gullotti, 1981). To prepare the title complex, the ligand BfbaK (1 mmol) was dissolved in methanol (10 ml) at 333 K and an aqueous solution (10 ml) containing Ni(AcO)_2_ . 4H_2_O (0.5 mmol) was added. The resulting solution was stirred at 333 K for 4 h, then cooled to room temperature and filtered. Blue crystals of the title complex suitable for X-ray diffraction were obtained by slow evaporation of this solution over a period of several days (yield 50%).

### Refinement

All H-atoms were placed in calculated positions with O—H = 0.85–0.87 Å and C—H = 0.93 Å and were allowed to ride in the refinement, with U</>_iso_(H) = 1.2U_eq_(C) or 1.5U_eq_(O).

### Crystal data

**Table:**

[Ni(C_12_H_9_N_2_O_3_S)_2_(H_2_O)_2_]·4H_2_O	*F*(000) = 1432
*M**_r_* = 689.35	*D*_x_ = 1.518 Mg m^−^^3^
Orthorhombic, *P**n**a*2_1_	Mo *K*α radiation, λ = 0.71073 Å
Hall symbol: P 2c -2n	Cell parameters from 5315 reflections
*a* = 13.865 (2) Å	θ = 2.1–25.1°
*b* = 11.5310 (18) Å	µ = 0.85 mm^−^^1^
*c* = 18.860 (3) Å	*T* = 296 K
*V* = 3015.3 (8) Å^3^	Prism, blue
*Z* = 4	0.36 × 0.19 × 0.14 mm

### Data collection

**Table:**

Bruker SMART CCD area-detector diffractometer	5315 independent reflections
Radiation source: fine-focus sealed tube	4983 reflections with *I* > 2σ(*I*)
Graphite monochromator	*R*_int_ = 0.028
φ and ω scans	θ_max_ = 25.1°, θ_min_ = 2.1°
Absorption correction: multi-scan (*SADABS*; Bruker, 2000)	*h* = −16→15
*T*_min_ = 0.821, *T*_max_ = 0.889	*k* = −12→13
16541 measured reflections	*l* = −21→22

### Refinement

**Table:**

Refinement on *F*^2^	Secondary atom site location: difference Fourier map
Least-squares matrix: full	Hydrogen site location: inferred from neighbouring sites
*R*[*F*^2^ > 2σ(*F*^2^)] = 0.034	H-atom parameters constrained
*wR*(*F*^2^) = 0.100	*w* = 1/[σ^2^(*F*_o_^2^) + (0.0792*P*)^2^ + 0.0306*P*] where *P* = (*F*_o_^2^ + 2*F*_c_^2^)/3
*S* = 0.96	(Δ/σ)_max_ = 0.001
5315 reflections	Δρ_max_ = 0.44 e Å^−^^3^
388 parameters	Δρ_min_ = −0.20 e Å^−^^3^
1 restraint	Absolute structure: Flack (1983), 2540 Friedel pairs
Primary atom site location: structure-invariant direct methods	Flack parameter: 0.00 (1)

### Special details

**Table:**

Geometry. All e.s.d.'s (except the e.s.d. in the dihedral angle between two l.s. planes) are estimated using the full covariance matrix. The cell e.s.d.'s are taken into account individually in the estimation of e.s.d.'s in distances, angles and torsion angles; correlations between e.s.d.'s in cell parameters are only used when they are defined by crystal symmetry. An approximate (isotropic) treatment of cell e.s.d.'s is used for estimating e.s.d.'s involving l.s. planes.
Refinement. Refinement of *F*^2^ against ALL reflections. The weighted *R*-factor *wR* and goodness of fit *S* are based on *F*^2^, conventional *R*-factors *R* are based on *F*, with *F* set to zero for negative *F*^2^. The threshold expression of *F*^2^ > σ(*F*^2^) is used only for calculating *R*-factors(gt) *etc*. and is not relevant to the choice of reflections for refinement. *R*-factors based on *F*^2^ are statistically about twice as large as those based on *F*, and *R*- factors based on ALL data will be even larger.

### Fractional atomic coordinates and isotropic or equivalent isotropic displacement parameters (Å^2^)

**Table:**

	*x*	*y*	*z*	*U*_iso_*/*U*_eq_	
Ni1	0.20498 (2)	1.02094 (3)	0.40723 (2)	0.02752 (11)	
S1	0.73596 (6)	1.04204 (7)	0.44399 (5)	0.03481 (19)	
S2	−0.05169 (7)	0.72312 (8)	0.70323 (5)	0.0424 (2)	
C1	0.1419 (2)	0.8284 (3)	0.51959 (18)	0.0344 (7)	
C2	0.0425 (3)	0.8359 (3)	0.51285 (19)	0.0416 (8)	
H2	0.0154	0.8610	0.4704	0.050*	
C3	−0.0162 (3)	0.8060 (3)	0.5690 (2)	0.0433 (8)	
H3	−0.0829	0.8099	0.5643	0.052*	
C4	0.0244 (3)	0.7701 (3)	0.63258 (18)	0.0367 (7)	
C5	0.1233 (3)	0.7666 (4)	0.64016 (19)	0.0446 (9)	
H5	0.1505	0.7449	0.6832	0.053*	
C6	0.1825 (3)	0.7957 (4)	0.5830 (2)	0.0439 (9)	
H6	0.2492	0.7930	0.5879	0.053*	
C7	0.2621 (3)	0.7887 (3)	0.4372 (2)	0.0383 (7)	
H7	0.2700	0.7168	0.4588	0.046*	
C8	0.3219 (2)	0.8221 (3)	0.37556 (19)	0.0345 (7)	
C9	0.3888 (3)	0.7478 (4)	0.3452 (2)	0.0517 (9)	
H9	0.3983	0.6740	0.3637	0.062*	
C10	0.4408 (3)	0.7847 (4)	0.2875 (2)	0.0613 (12)	
H10	0.4874	0.7369	0.2673	0.074*	
C11	0.4233 (3)	0.8925 (4)	0.2600 (2)	0.0554 (11)	
H11	0.4573	0.9190	0.2207	0.067*	
C12	0.3539 (3)	0.9611 (3)	0.2919 (2)	0.0407 (8)	
H12	0.3417	1.0342	0.2730	0.049*	
C13	0.4683 (2)	1.0089 (3)	0.52834 (19)	0.0393 (8)	
H13	0.4397	0.9777	0.5687	0.047*	
C14	0.5673 (3)	1.0014 (3)	0.5188 (2)	0.0399 (8)	
H14	0.6053	0.9657	0.5530	0.048*	
C15	0.6096 (2)	1.0470 (3)	0.45836 (18)	0.0335 (7)	
C16	0.5529 (2)	1.0996 (3)	0.4065 (2)	0.0369 (7)	
H16	0.5814	1.1292	0.3657	0.044*	
C17	0.4550 (2)	1.1077 (3)	0.41573 (19)	0.0356 (7)	
H17	0.4171	1.1426	0.3811	0.043*	
C18	0.4121 (2)	1.0635 (3)	0.47707 (18)	0.0316 (6)	
C19	0.2719 (2)	1.1073 (3)	0.54054 (19)	0.0383 (8)	
H19	0.3101	1.1220	0.5801	0.046*	
C20	0.1660 (2)	1.1226 (3)	0.5437 (2)	0.0390 (8)	
C21	0.1207 (3)	1.1581 (6)	0.6040 (3)	0.0727 (16)	
H21	0.1552	1.1711	0.6455	0.087*	
C22	0.0210 (3)	1.1745 (6)	0.6016 (3)	0.0765 (16)	
H22	−0.0121	1.1990	0.6418	0.092*	
C23	−0.0268 (3)	1.1544 (4)	0.5405 (2)	0.0517 (10)	
H23	−0.0932	1.1646	0.5382	0.062*	
C24	0.0239 (2)	1.1186 (3)	0.4818 (2)	0.0399 (8)	
H24	−0.0093	1.1060	0.4397	0.048*	
N1	0.20060 (18)	0.8593 (2)	0.45990 (15)	0.0318 (6)	
N2	0.30403 (16)	0.9275 (2)	0.34813 (15)	0.0292 (6)	
N3	0.30951 (17)	1.0737 (2)	0.48275 (15)	0.0297 (6)	
N4	0.11907 (18)	1.1011 (2)	0.48314 (14)	0.0318 (6)	
O1	0.22444 (18)	1.1677 (2)	0.34556 (13)	0.0394 (5)	
H1A	0.2096	1.1876	0.3036	0.059*	
H1	0.2364	1.2285	0.3694	0.059*	
O2	0.09435 (18)	0.9625 (2)	0.34255 (14)	0.0463 (6)	
H2B	0.0449	1.0005	0.3295	0.069*	
H2A	0.1204	0.9324	0.3061	0.069*	
O3	0.77809 (19)	0.9764 (2)	0.50163 (16)	0.0438 (6)	
O4	0.76778 (18)	1.1617 (2)	0.44222 (19)	0.0562 (7)	
O5	0.7506 (2)	0.9857 (3)	0.37643 (17)	0.0624 (8)	
O6	0.0109 (2)	0.7141 (3)	0.76410 (16)	0.0728 (9)	
O7	−0.1269 (2)	0.8077 (3)	0.71181 (17)	0.0644 (8)	
O8	−0.0890 (3)	0.6119 (3)	0.68130 (19)	0.0814 (12)	
O1W	0.1482 (2)	0.8187 (3)	0.23302 (17)	0.0613 (8)	
H1WA	0.1788	0.7548	0.2339	0.092*	
H1WB	0.1766	0.8647	0.2047	0.092*	
O2W	0.2203 (3)	−0.0066 (3)	0.1507 (2)	0.0764 (10)	
H2WA	0.2499	0.0164	0.1126	0.115*	
H2WB	0.1642	0.0260	0.1517	0.115*	
O4W	0.1939 (3)	0.5819 (3)	0.2341 (2)	0.0784 (10)	
H4WB	0.1951	0.5164	0.2134	0.118*	
H4WA	0.2094	0.5718	0.2775	0.118*	
O3W	0.9288 (2)	0.0644 (4)	0.3082 (2)	0.0940 (13)	
H3WB	0.9386	0.1276	0.2860	0.141*	
H3WA	0.8695	0.0599	0.3187	0.141*	

### Atomic displacement parameters (Å^2^)

**Table:**

	*U*^11^	*U*^22^	*U*^33^	*U*^12^	*U*^13^	*U*^23^
Ni1	0.02597 (19)	0.0301 (2)	0.0265 (2)	0.00072 (14)	0.00129 (17)	0.00007 (19)
S1	0.0260 (4)	0.0403 (4)	0.0381 (4)	0.0023 (3)	−0.0034 (4)	0.0044 (4)
S2	0.0496 (5)	0.0457 (5)	0.0319 (4)	−0.0013 (4)	0.0112 (4)	0.0058 (4)
C1	0.0388 (17)	0.0286 (16)	0.0359 (18)	−0.0041 (13)	0.0049 (14)	0.0026 (13)
C2	0.0422 (18)	0.053 (2)	0.0296 (18)	−0.0063 (16)	−0.0020 (14)	0.0095 (15)
C3	0.0361 (17)	0.057 (2)	0.0371 (19)	−0.0087 (16)	0.0010 (15)	0.0071 (16)
C4	0.0438 (18)	0.0354 (17)	0.0308 (17)	−0.0037 (15)	0.0077 (14)	0.0043 (13)
C5	0.044 (2)	0.056 (2)	0.0336 (19)	0.0032 (17)	−0.0003 (15)	0.0141 (16)
C6	0.0357 (17)	0.055 (2)	0.041 (2)	0.0057 (16)	0.0024 (15)	0.0056 (17)
C7	0.0445 (17)	0.0305 (16)	0.0398 (18)	0.0017 (14)	0.0043 (15)	0.0059 (15)
C8	0.0350 (16)	0.0336 (18)	0.0348 (16)	0.0045 (14)	0.0023 (14)	−0.0009 (14)
C9	0.051 (2)	0.050 (2)	0.054 (2)	0.0147 (18)	0.0089 (18)	0.0039 (19)
C10	0.057 (2)	0.070 (3)	0.057 (3)	0.026 (2)	0.021 (2)	0.000 (2)
C11	0.047 (2)	0.076 (3)	0.043 (2)	0.002 (2)	0.0176 (17)	0.003 (2)
C12	0.0419 (19)	0.042 (2)	0.038 (2)	−0.0046 (15)	0.0028 (15)	0.0034 (15)
C13	0.0344 (19)	0.048 (2)	0.0360 (19)	0.0017 (14)	0.0031 (15)	0.0152 (15)
C14	0.0326 (18)	0.051 (2)	0.036 (2)	0.0058 (15)	−0.0025 (15)	0.0153 (15)
C15	0.0310 (16)	0.0329 (16)	0.0366 (19)	−0.0008 (13)	−0.0035 (13)	0.0025 (14)
C16	0.0287 (14)	0.0475 (18)	0.0345 (16)	−0.0057 (12)	−0.0021 (15)	0.0108 (17)
C17	0.0296 (14)	0.0442 (18)	0.0330 (17)	−0.0031 (12)	−0.0049 (14)	0.0097 (15)
C18	0.0263 (14)	0.0344 (16)	0.0340 (17)	−0.0036 (13)	−0.0017 (13)	0.0031 (13)
C19	0.0304 (16)	0.056 (2)	0.0288 (18)	−0.0049 (15)	−0.0009 (13)	−0.0048 (15)
C20	0.0333 (17)	0.048 (2)	0.0353 (18)	−0.0010 (15)	−0.0001 (14)	−0.0068 (15)
C21	0.038 (2)	0.137 (5)	0.043 (2)	0.002 (2)	0.0019 (18)	−0.031 (3)
C22	0.041 (2)	0.130 (5)	0.059 (3)	0.001 (3)	0.014 (2)	−0.039 (3)
C23	0.0325 (18)	0.068 (3)	0.055 (2)	0.0020 (16)	0.0051 (17)	−0.014 (2)
C24	0.0313 (16)	0.0436 (18)	0.045 (2)	0.0031 (14)	−0.0040 (15)	−0.0055 (15)
N1	0.0340 (14)	0.0281 (13)	0.0332 (15)	−0.0024 (10)	0.0056 (10)	0.0037 (11)
N2	0.0262 (12)	0.0304 (15)	0.0309 (15)	−0.0020 (10)	0.0041 (10)	−0.0031 (11)
N3	0.0272 (12)	0.0332 (14)	0.0287 (14)	−0.0021 (10)	−0.0006 (11)	0.0004 (11)
N4	0.0301 (13)	0.0322 (14)	0.0331 (15)	0.0001 (10)	0.0037 (11)	−0.0023 (11)
O1	0.0539 (13)	0.0300 (12)	0.0342 (13)	0.0023 (10)	−0.0040 (11)	0.0049 (10)
O2	0.0380 (13)	0.0588 (16)	0.0421 (15)	0.0019 (11)	−0.0068 (11)	−0.0094 (12)
O3	0.0369 (13)	0.0453 (15)	0.0491 (17)	0.0051 (10)	−0.0089 (11)	0.0065 (12)
O4	0.0345 (12)	0.0447 (15)	0.089 (2)	−0.0049 (11)	−0.0096 (14)	0.0229 (16)
O5	0.0443 (17)	0.097 (2)	0.0458 (17)	0.0119 (15)	0.0030 (14)	−0.0110 (17)
O6	0.075 (2)	0.107 (3)	0.0359 (16)	−0.002 (2)	0.0060 (14)	0.0233 (17)
O7	0.0660 (18)	0.077 (2)	0.0499 (18)	0.0180 (15)	0.0253 (15)	0.0135 (16)
O8	0.110 (3)	0.061 (2)	0.073 (2)	−0.0346 (18)	0.047 (2)	−0.0115 (16)
O1W	0.0578 (17)	0.0614 (18)	0.065 (2)	−0.0034 (14)	0.0028 (14)	−0.0054 (15)
O2W	0.096 (3)	0.081 (2)	0.052 (2)	0.0126 (19)	0.0092 (17)	0.0085 (18)
O4W	0.100 (3)	0.062 (2)	0.073 (2)	−0.0125 (18)	−0.0150 (19)	0.0097 (18)
O3W	0.0498 (19)	0.129 (3)	0.103 (3)	0.015 (2)	0.0142 (19)	0.046 (3)

### Geometric parameters (Å, º)

**Table:**

Ni1—N2	2.071 (3)	C12—H12	0.9300
Ni1—O1	2.071 (2)	C13—C14	1.387 (5)
Ni1—O2	2.073 (3)	C13—C18	1.392 (5)
Ni1—N4	2.079 (3)	C13—H13	0.9300
Ni1—N1	2.113 (3)	C14—C15	1.385 (5)
Ni1—N3	2.121 (3)	C14—H14	0.9300
S1—O5	1.444 (3)	C15—C16	1.393 (5)
S1—O3	1.448 (3)	C16—C17	1.371 (4)
S1—O4	1.449 (3)	C16—H16	0.9300
S1—C15	1.774 (3)	C17—C18	1.397 (5)
S2—O7	1.437 (3)	C17—H17	0.9300
S2—O8	1.443 (3)	C18—N3	1.431 (4)
S2—O6	1.443 (3)	C19—N3	1.269 (5)
S2—C4	1.784 (3)	C19—C20	1.480 (5)
C1—C6	1.375 (5)	C19—H19	0.9300
C1—C2	1.387 (5)	C20—N4	1.338 (5)
C1—N1	1.434 (4)	C20—C21	1.362 (6)
C2—C3	1.380 (5)	C21—C22	1.396 (6)
C2—H2	0.9300	C21—H21	0.9300
C3—C4	1.387 (5)	C22—C23	1.349 (6)
C3—H3	0.9300	C22—H22	0.9300
C4—C5	1.379 (5)	C23—C24	1.375 (5)
C5—C6	1.395 (5)	C23—H23	0.9300
C5—H5	0.9300	C24—N4	1.335 (4)
C6—H6	0.9300	C24—H24	0.9300
C7—N1	1.254 (4)	O1—H1A	0.8499
C7—C8	1.479 (5)	O1—H1	0.8500
C7—H7	0.9300	O2—H2B	0.8500
C8—N2	1.344 (4)	O2—H2A	0.8500
C8—C9	1.386 (5)	O1W—H1WA	0.8506
C9—C10	1.374 (6)	O1W—H1WB	0.8501
C9—H9	0.9300	O2W—H2WA	0.8684
C10—C11	1.369 (6)	O2W—H2WB	0.8637
C10—H10	0.9300	O4W—H4WB	0.8501
C11—C12	1.384 (6)	O4W—H4WA	0.8538
C11—H11	0.9300	O3W—H3WB	0.8511
C12—N2	1.324 (4)	O3W—H3WA	0.8476
			
N2—Ni1—O1	92.09 (10)	N2—C12—H12	118.5
N2—Ni1—O2	90.27 (10)	C11—C12—H12	118.5
O1—Ni1—O2	91.81 (10)	C14—C13—C18	119.4 (3)
N2—Ni1—N4	169.04 (11)	C14—C13—H13	120.3
O1—Ni1—N4	95.63 (10)	C18—C13—H13	120.3
O2—Ni1—N4	97.23 (11)	C15—C14—C13	120.1 (3)
N2—Ni1—N1	79.24 (11)	C15—C14—H14	119.9
O1—Ni1—N1	171.32 (10)	C13—C14—H14	119.9
O2—Ni1—N1	88.20 (10)	C14—C15—C16	120.2 (3)
N4—Ni1—N1	92.98 (11)	C14—C15—S1	122.1 (3)
N2—Ni1—N3	93.30 (10)	C16—C15—S1	117.6 (3)
O1—Ni1—N3	93.09 (10)	C17—C16—C15	119.9 (3)
O2—Ni1—N3	173.83 (11)	C17—C16—H16	120.0
N4—Ni1—N3	78.55 (10)	C15—C16—H16	120.0
N1—Ni1—N3	87.53 (11)	C16—C17—C18	120.1 (3)
O5—S1—O3	111.75 (17)	C16—C17—H17	120.0
O5—S1—O4	111.4 (2)	C18—C17—H17	120.0
O3—S1—O4	113.12 (18)	C13—C18—C17	120.1 (3)
O5—S1—C15	106.73 (18)	C13—C18—N3	122.8 (3)
O3—S1—C15	107.49 (16)	C17—C18—N3	117.1 (3)
O4—S1—C15	105.88 (15)	N3—C19—C20	118.6 (3)
O7—S2—O8	112.0 (2)	N3—C19—H19	120.7
O7—S2—O6	113.3 (2)	C20—C19—H19	120.7
O8—S2—O6	112.3 (2)	N4—C20—C21	123.0 (3)
O7—S2—C4	107.90 (17)	N4—C20—C19	115.2 (3)
O8—S2—C4	105.55 (18)	C21—C20—C19	121.8 (3)
O6—S2—C4	105.09 (17)	C20—C21—C22	118.1 (4)
C6—C1—C2	120.2 (3)	C20—C21—H21	120.9
C6—C1—N1	121.3 (3)	C22—C21—H21	120.9
C2—C1—N1	118.5 (3)	C23—C22—C21	119.3 (4)
C3—C2—C1	120.0 (3)	C23—C22—H22	120.3
C3—C2—H2	120.0	C21—C22—H22	120.3
C1—C2—H2	120.0	C22—C23—C24	119.2 (3)
C2—C3—C4	119.9 (3)	C22—C23—H23	120.4
C2—C3—H3	120.1	C24—C23—H23	120.4
C4—C3—H3	120.1	N4—C24—C23	122.3 (3)
C5—C4—C3	120.2 (3)	N4—C24—H24	118.8
C5—C4—S2	120.1 (3)	C23—C24—H24	118.8
C3—C4—S2	119.7 (3)	C7—N1—C1	119.5 (3)
C4—C5—C6	119.8 (3)	C7—N1—Ni1	113.1 (2)
C4—C5—H5	120.1	C1—N1—Ni1	127.2 (2)
C6—C5—H5	120.1	C12—N2—C8	118.5 (3)
C1—C6—C5	119.8 (3)	C12—N2—Ni1	128.7 (2)
C1—C6—H6	120.1	C8—N2—Ni1	112.7 (2)
C5—C6—H6	120.1	C19—N3—C18	119.9 (3)
N1—C7—C8	118.7 (3)	C19—N3—Ni1	112.6 (2)
N1—C7—H7	120.7	C18—N3—Ni1	127.2 (2)
C8—C7—H7	120.7	C20—N4—C24	118.0 (3)
N2—C8—C9	121.5 (3)	C20—N4—Ni1	113.0 (2)
N2—C8—C7	115.8 (3)	C24—N4—Ni1	128.3 (2)
C9—C8—C7	122.6 (3)	Ni1—O1—H1A	135.5
C10—C9—C8	119.2 (4)	Ni1—O1—H1	113.7
C10—C9—H9	120.4	H1A—O1—H1	108.5
C8—C9—H9	120.4	Ni1—O2—H2B	126.8
C9—C10—C11	119.2 (4)	Ni1—O2—H2A	107.1
C9—C10—H10	120.4	H2B—O2—H2A	108.6
C11—C10—H10	120.4	H1WA—O1W—H1WB	108.8
C10—C11—C12	118.6 (4)	H2WA—O2W—H2WB	108.2
C10—C11—H11	120.7	H4WB—O4W—H4WA	108.3
C12—C11—H11	120.7	H3WB—O3W—H3WA	108.7
N2—C12—C11	123.0 (3)		

### Hydrogen-bond geometry (Å, º)

**Table:**

*D*—H···*A*	*D*—H	H···*A*	*D*···*A*	*D*—H···*A*
O1—H1···O4^i^	0.85	1.92	2.748 (4)	166
O1—H1*A*···O7^ii^	0.85	2.08	2.876 (4)	156
O2—H2*A*···O1*W*	0.85	1.94	2.752 (4)	159
O2—H2*B*···O3*W*^iii^	0.85	1.82	2.659 (4)	171
O1*W*—H1*WA*···O4*W*	0.85	2.00	2.804 (5)	156
O1*W*—H1*WB*···O2*W*^iv^	0.85	1.90	2.733 (5)	167
O2*W*—H2*WA*···O3^v^	0.87	2.13	2.833 (5)	137
O2*W*—H2*WB*···O7^vi^	0.86	2.29	2.874 (5)	125
O3*W*—H3*WB*···O6^v^	0.85	2.00	2.813 (6)	160
O3*W*—H3*WA*···O5^vii^	0.85	2.15	2.930 (5)	152
O4*W*—H4*WB*···O8^vi^	0.85	2.17	2.846 (5)	136
O4*W*—H4*WA*···O5^viii^	0.85	2.06	2.903 (5)	169

Symmetry codes: (i) *x*−1/2, −*y*+5/2, *z*; (ii) −*x*, −*y*+2, *z*−1/2; (iii) *x*−1, *y*+1, *z*; (iv) *x*, *y*+1, *z*; (v) −*x*+1, −*y*+1, *z*−1/2; (vi) −*x*, −*y*+1, *z*−1/2; (vii) *x*, *y*−1, *z*; (viii) *x*−1/2, −*y*+3/2, *z*.
